# ReGeneraTing Agents (RGTA^®^): A new option for healing and improving treatment outcomes for traumatic and burn injuries of the hand

**DOI:** 10.1002/ccr3.2054

**Published:** 2019-02-17

**Authors:** Sharifah Ahmad Roohi, Denis Barritault

**Affiliations:** ^1^ Department of Orthopaedics, Faculty of Medicine and Health Sciences Universiti Putra Malaysia Serdang Selangor Darul Ehsan Malaysia; ^2^ Hand & Upper Limb Centre Pantai Hospital Kuala Lumpur Kuala Lumpur Malaysia; ^3^ OTR3 (Organe, Tissue, Régénération, Réparation, Remplacement) Paris France; ^4^ Laboratory Cell Growth and Tissue Repair (CRRET), UPEC 4397/ERL, CNRS 9215 Université‐Paris‐Est‐Créteil Créteil France

**Keywords:** CACIPLIQ, finger, functional recovery, heparan sulfate mimetic, matrix therapy

## Abstract

CACIPLIQ20^®^ significantly improved the outcomes of severe burn injuries of the hand. Healing was accelerated, with little or no scarring, allowing for greater mobility over the joints and maintained suppleness. Functional recovery was achieved in all cases.

## 1 INTRODUCTION

Four patients with traumatic hand burns were treated with CACIPLIQ20^®^, a matrix therapy based on ReGeneraTing Agents. Range of motion was restored, healing by secondary intention occurred, and pain of dressing was reduced. CACIPLIQ20^®^ can be used at earlier or even later stages to prevent an adverse outcome.

In the current day practice of hand surgery, achieving good outcomes for emergency cases presenting with significant trauma and soft tissue injury, or for cases with complications from initial treatments remains a challenge. We had the opportunity in our clinic to use a type of matrix therapy, commercially known as CACIPLIQ20^®^, which is recommended to treat chronic wounds.^1^, ^2^ Matrix therapy using RGTA^®^ (ReGeneraTing Agent) is an innovative, minimally invasive approach in the field of regenerative medicine that aims to promote tissue regeneration by reconstructing the cellular microenvironment following tissue injuries.^3^ CACIPLIQ20^®^ contains OTR4120, a RGTA^®^ specifically formulated for skin and plastic surgery, which replaces degraded Heparan Sulfate (HS) in the injured extracellular matrix.^4^, ^5^

Generally speaking, vascularity of the upper extremity is usually not an issue, especially when compared to the lower extremity. However, in cases of full‐thickness skin loss (FTSL) due to burns, the wound bed is not vascularized well and is thus prone to infection. Consequently, skin and tissue coverage become an issue, especially if it is over a joint. In the hand, wound healing often leads to scar contractures and is a highly undesirable complication. Therefore, in order to prevent scar contracture and increase the patient's chance for recovery, improving, and speeding up the wound healing process is of utmost importance.

Here, we present four cases treated from 2010 where CACIPLIQ20^®^ was initially used as a last resort to avoid a dismal outcome and then was used progressively at earlier stages in order to improve functionality. The four selected cases include patients with different backgrounds and burns of varying causes. All were treated with CACIPLIQ20^®^ after the initial conventional treatment methods failed or no alternative acceptable treatment was found. These cases would have otherwise resulted in a poor outcome with significant scarring causing contractures or stiffness and/or may have required a more proximal amputation.

## 2 METHODS

### 2.1 Patients

All four cases were chosen after a specific surgical index procedure was performed in the emergency setting where the wounds were either present primarily or developed secondarily due to failure of the conventional treatment (debridement, antibiotics, and dressing) in the primary setting. CACIPLIQ20^®^ was applied in the described fashion (see “intervention” section below) to either compromised patients (diabetic, chronic smokers) or ischemic wounds (amputations, full‐thickness loss) once the wounds were found to be not healing or regressing.

### 2.2 Materials

CACIPLIQ20^®^ is the commercial name for a skin‐adapted RGTA^®^‐based product to treat chronic wounds. RGTA^®^s are chemically engineered polymers that are specifically designed to replace degraded Heparan Sulfate (HS) in the injured extracellular matrix. They protect naturally existing structural and signaling proteins by sequestering proteins through low‐affinity binding, allowing for faster and high‐quality tissue repair.^3^ Their unique properties have been the subject of intensive preclinical and clinical studies.^1^, ^2^, ^5^, ^6^, ^7^, ^8^, ^9^, ^10^, ^11^, ^12^, ^13^, ^14^ CACIPLIQ20^®^ contains RGTA^®^ OTR4120, a biodegradable polymer of 1‐6 alpha polyglucose substituted carboxymethyl and sulfated.^3^

### 2.3 Intervention

CACIPLIQ20^®^ was applied twice a week by soaking a sterile gauze with the solution and placing it on the wound for 12 minutes. The gauze was then removed, and the wound was covered with a non‐occlusive dressing. When debridement was required, local antibiotics and appropriate dressing materials were substituted or used in conjunction before or after the intervention.

## 3 RESULTS

### 3.1 Case reports: Burns

A total of four male patients (42.25 ± 17.52 years old) were addressed to the hospital with burns that led to full‐thickness skin graft. The clinical data are summarized in Table 1 and described below.

**Table 1:** Summary of FTS loss and burn cases (n = 4)

Case #	Age/gender	Type of lesion	Smoker	History & Healing by primary or secondary intention	Infection (Y/N)	Ischemia (Y/N)	Start of CCPL (days)	Estimated success evaluation
1	52/M	IF & MF 3rd degree fire burns	Y moderate	Secondary; total AMP of MF and of P3 IF	Y	Y, Thrombosis of both vessels	POD21	AMP avoided
2	51/M	Chemical cautery of granulation on RF	Y heavy	Secondary; after cautery by CuSO4	N	N	POD14	Wound healed in 24 d
3	50/M	Friction (abrasion) burn	N	Primary; graft stagnant after 10 d	N	N	POD11	Skin grafting, complete healing & pain reduction in 12 days
4	16/M	RF 3rd degree electrical burn	N	Secondary; topical application of honey & oral Aug‐ mentin	Y	N	POD21	Pain relief, rapid healing, & reduced scarring with excellent mobility pre‐ served over joint

AB, antibiotic; AMP, amputation; CCPL, CACIPLIQ 20^®^; DM, diabetes mellitus; FTSG, full thickness skin Graft; IF, index finger; MF, middle finger; P1, proximal phalanx; P2, middle phalanx; P3, distal phalanx; POD, postoperative day; RF, ring finger; SF, small finger.

### 3.2 Patient 1. High voltage electrical burn

**Figure 1:**
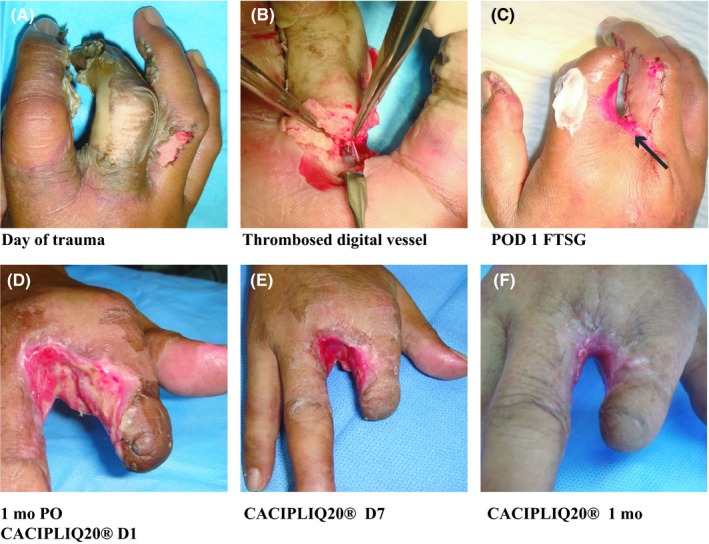
High voltage electrical burn. A, Patient burnt his right hand, sustaining ulnar‐sided burns to his ring finger and thumb (full thickness). The whole middle finger was completely burnt as was the index up to the PIP joint. B, The complete destruction of the soft tissues with thrombosis of both digital vessels prompted the decision to amputate the MF through the MCP joint. C, A FTSG is applied to the RF (arrow) after an amputation of the MF and part of the index finger (through the PIP joint) was performed. D, Complete loss of the graft with increasing slough even a month after the injury prompted the use of CACIPLIQ20^®^. E, Within a week of starting CACIPLIQ 20^®^, the wound was converted to a clean, contracting one. F, 1 mo after CACIPLIQ20^®^, the wound had healed completely

Patient 1, a 52‐year‐old right‐handed technician with the local power company was referred to as an emergency case after his right hand was burnt. He had used his hand to shield his face from a fire which exploded while he was repairing an 11 000 V electrical cable. He sustained ulnar‐sided full‐thickness burns to his ring finger and thumb. The whole middle finger was completely burnt, including the index finger up to the PIP joint (Figure 1A). An amputation of the middle finger (through the MCP joint) and part of the index finger (through the PIP joint) was performed after viewing the complete thrombosis of both digital vessels and complete destruction of the soft tissues (Figure 1B). The patient refused any subsequent surgery and insisted on conservative management for up to 1 month (Figure 1C). Loss of the skin graft, persistent wounds, and poor healing prompted the use of CACIPLIQ20^®^ (Figure 1D). Improvement with granulation tissue formation and pain reduction were seen within a week (Figure 1E). After a month, the wounds had dried and healing by secondary intention had taken place (Figure 1F). There was some syndactyly between the index and ring fingers with some limitation of movement.

#### 3.2.1 Discussion

Patient 1 suffered a severe third‐degree burn to his right (dominant) hand, which at presentation required amputation and debridement. The patient refused surgical treatment after the index procedure, and he had a large web space wound (measuring 40 by 60 mm) with some slough (Figure 1B,C). The wound contracted to less than half of its original size within the first week of CACIPLIQ20^®^ application, a testament to the regenerative agent's capabilities (Figure 1E). Finally, healing of the second web space resulted in some contracture. However, full range of movement of the index finger was achieved (metacarpophalangeal MP joint), which is unusual in burn contracture cases (Figure 1F).

### 3.3 Patient 2. Chemical cautery of granulation tissue

**Figure 2:**
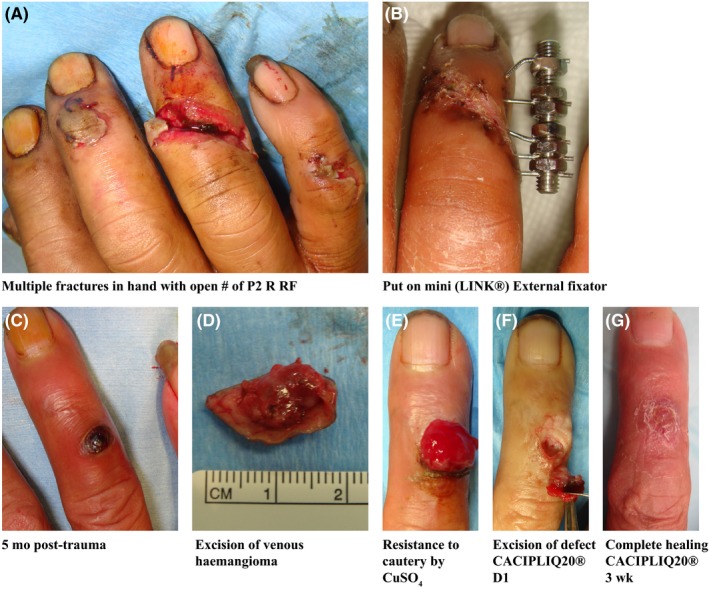
Chemical cautery of granulation tissue. A, An outstation lorry business owner sustained an open fracture of his R RF as part of multiple injuries to his right hand after it was hit by a steel tire rim. B, A mini external fixator was applied, and it went on to heal. C, 5 mo post‐trauma, he developed a venous hemangioma. D, This was excised with clear margins. E, Excessive granulation tissue occupied the defect and was resistant to cautery by copper sulfate (CuSO4). F, Excision was done, and the 4 mm deep defect treated with CACIPLIQ 20^®^. G, Complete healing in 3 wk

Patient 2, a 51‐year‐old truck company owner was installing a tire rim when the tire blew and the rim hit his right hand. The small circular steel frame inflicted multiple injuries corresponding to its circumferential shape—distal radius fracture, 5th metacarpal fracture, open injury to PIP joint of small finger, fracture middle phalanx of Ring finger (Figure 2A,B), middle fingertip injury, and thumb proximal phalanx fracture. All were fixed with wires and external fixators according to the patient's wishes, and all went on to heal. The External fixators were removed 2 months later, and he was discharged a month after that.

Six weeks later (5 months post‐trauma), he called in to report a “growth” (Figure 2C) which was diagnosed as a hemangioma. This probably resulted from the fracture healing process or from the insertion of k‐wires/external fixator wires causing a veno‐capillary fusion/proliferation. After 3 months of observation, an excision was performed, since the hemangioma had grown in size and the margins were reported as free of tumor—lesion 1‐2 mm away from edge (Figure 2D). Six weeks after excision, the patient called to say the lesion had returned. However, it was actually granulation tissue extruding from the wound (Figure 2E). After application of copper sulfate to cauterize the granulation, it was completely excised and left a cavity measuring 10 mm by 8 mm and a depth of 4 mm (Figure 2F). To avoid the reoccurrence of the same healing complications, CACIPLIQ20^®^ was administered to the defect and within 3 weeks healed completely with a good range of motion (Figure 2G).

#### 3.3.1 Discussion

Patient 2 developed two minor complications of his injury, which persisted despite repeated usage of copper sulfate crystals to ablate the re‐occurring hyper‐granulation. Thus, we decided to use a regenerating agent to help improve the healing process and prevent the recurrence of hyper‐granulation. Although it was a relatively small wound after excision (Figure 2F), the patient being a heavy smoker (30‐40 cigarettes per day) was of concern. Despite these unfavorable factors, complete healing took place in 3 weeks, dorsal skin suppleness was maintained and a functional range of motion was achieved.

### 3.4 Patient 3. Friction burn of the palm

**Figure 3:**
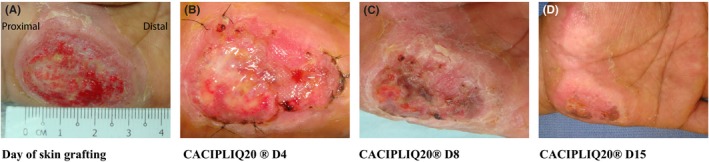
Friction burn of the palm. A, Patient 3 had a motorcycle accident and grazed his palm along the road to break his fall sustaining a deep full‐thickness friction burn up to and including the fat pad of his hypothenar eminence measuring 40 mm by 30 mm. B, CACIPLIQ20^®^ was started after a failed skin graft starting with a 33 × 25 mm sized wound. C, Within 1 wk, the wound dried up and reduced by half in size. D, Two weeks later the wound had dried up

Patient 3, a 50‐year‐old gentleman, had a motorcycle accident and used his left hand to break his fall, grazing his palm along the road. He sustained a deep full‐thickness friction burn up to and including the fat pad of the hypothenar eminence, measuring 40 mm by 30 mm (Figure 3A). After initial daily dressings for 6 days, which he found excruciatingly painful, skin grafting was performed at his request. Unfortunately, after 10 days the graft did not take and sloughed off leaving a 33 × 25 mm sized wound (Figure 3B), which would have taken at least 4 weeks to heal.^15^ It was decided to start CACIPLIQ20^®^ the next day because his pain tolerance was low (VAS—visual analog score—of 7) and conventional treatments had failed. One week after application, improvement (Figure 3C) and pain relief were already felt by the patient. Within 2 weeks of commencing CACIPLIQ20^®^, the patient's VAS score dropped from 7 to 4 and the wound size had reduced to 5 mm by 5 mm (Figure 3D). All of his wounds dried up by day 17 of application.

#### 3.4.1 Discussion

Patient 3 had a full‐thickness wound (Figure 3A) measuring 30 mm by 40 mm at presentation which was treated both by conventional dressings and by skin grafting, partly because of his low threshold of pain. When the graft did not take (Figure 3B), CACIPLIQ20^®^ was started. A dramatic improvement in both size and pain level was achieved, contrary to the results with previous initial treatments. The wound size was reduced by half in 1 week (Figure 3C) and almost healed by 2 weeks (Figure 3D), unexpectedly showing an accelerated rate of healing.

### 3.5 Patient 4. Electrical burn

**Figure 4:**
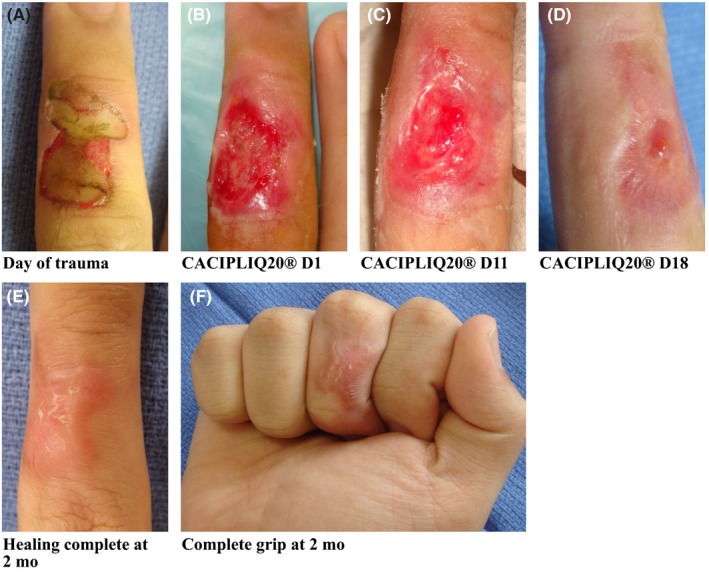
Electrical burn. A, Patient 4 was electrocuted instantly on the radial three digits of his right hand, with third‐degree burns to the dorsum of his MF measuring 20 mm × 10 mm. B, CACIPLIQ20^®^ was started 24 d after the trauma occurred. C, Healing was observed within a week as seen by the formulation of granulation tissue and by Day 11 the wound was half its size. D, Wound closed by D18. E and F, Healing was complete and full range of motion with strong grip was achieved by 2 mo of application

Patient 4, a right hand‐dominant 16‐year‐old student was doing an experiment in his school laboratory using a test probe in water. He accidentally touched the electrical probe and was electrocuted instantly on the radial three digits of his right hand. His thumb and index finger only had blisters but the dorsum of his middle finger (MF) sustained third‐degree burns measuring 20 mm × 10 mm (Figure 4A). He was brought to the hospital 2 days later, and an Actilite^®^ (honey) dressing was applied. Two weeks later he re‐injured it slightly, and the dressing was wet at presentation. At 3 weeks post trauma, the wound was dirty with slough, so he underwent a debridement and Augmentin was started. It was decided to start him on CACIPLIQ20^®^ at that time (Figure 4B) to help enhance the healing process. The healing process was rapid and observed within a week, with granulation tissue covering half the wound by day 11 (Figure 4C). Almost complete healing was achieved by 18 days (Figure 4D). Two months post application the wound had healed beautifully. The patient regained a full range of motion and grip, which in cases of healing by scar formation generally results in contracture and stiffness (Figure 4E,F).

#### 3.5.1 Discussion

Patient 4 was a young patient whose healing was not progressing as expected, although the damage sustained by electrical burns may be difficult to evaluate. CACIPLIQ20^®^ was started both to manage the pain during dressings and to reduce the size of the expected contracture while maintaining the suppleness of the dorsal skin. Although speedy healing was expected due to the young age of the patient, his lack of co‐morbidities, and the small size of the wound (10 mm by 20 mm), the application of the regenerating agent allowed pain relief with rapid healing, reduced scarring, and preserved excellent mobility of the PIP joint (Figure 4F).

## 4 DISCUSSION

Rapid healing was observed in all four cases, with the most dramatic effect in the first patient. More specifically, after dressing the wound for a month and it regressing, healing with wound contracture of 50% took place within a week of application. The skin quality was very much native to the defect and excellent over the joints, allowing for complete range of motion with hardly any contracture or scarring observed.

Notably, all patients experienced remarkable pain relief shortly after CACIPLIQ20^®^ application. This added benefit is especially important, since it greatly affects patients’ quality of life while they are trying to heal from severe and traumatic injury.

Two out of the four presented cases received skin grafts which did not take and only started healing after CACIPLIQ20^®^ treatment began. Thus, it would be of great benefit to use CACIPLIQ20^®^ in first intention at the same time as the skin grafting procedure, in order to potentially improve the outcome of the graft, or in minor cases to avoid use of the graft altogether. Although very promising, these results reflect a small number of cases. It would be necessary to perform a more thorough evaluation of CACIPLIQ20^®^’s safety and efficacy, preferably through a double‐blinded RCT.

It is important to note that thorough debridement is a prerequisite when using CACIPLIQ20^®^. Although wounds with mild infection or slough did heal, healing was more efficient if debridement was performed. Additionally, an infection has to be cleared with either local or systemic antibiotics before initiating treatment. Moreover, approximation of tissue aided by supportive tapes enhanced the process. Finally, various dressings may need to be used as an adjunct to deal with the different types of wounds.

In conclusion, RGTA^®^ therapy helped speed up the healing process in severe burn wounds. Advantages include little or no scarring, allowing greater mobility over joints and maintaining suppleness. An added benefit was pain relief. A larger study should be performed to confirm these results, as well as assess the effect on nerve and tendon healing.

## CONFLICT OF INTEREST

DB has financial interest as inventor of the patented RGTA^®^ technology.

## AUTHOR CONTRIBUTIONS

All authors have made substantial contributions to all of the following: SAR: involved in the conception and design of the study, acquisition of data, analysis, and interpretation of data. SAR and DB: drafted the article, revised it critically for important intellectual content and approved the final version for submission.

## ETHICS AND RESEARCH APPROVAL

The patients provided written informed consent as per the Hospital's institutional review board guidelines. As CACIPLIQ20^®^ is available on the market as a healing agent and only individual cases are reported; as such, no specific authorization was requested.
